# Epidemiology of Bronchiolitis in Hospitalized Infants at Tawam Hospital, Al Ain, United Arab Emirates

**DOI:** 10.2174/1874306402115010007

**Published:** 2021-05-24

**Authors:** Amar Al Shibli, Muhammad B. Nouredin, Abdulla Al Amri, Durdana Iram, Hassib Narchi

**Affiliations:** 1 Department of Pediatrics, Tawam Hospital, Al-Ain, Abu Dhabi, United Arab Emirates; 2 Department of Pediatrics, Faculty of Medicine and Health Sciences, Abu Dhabi, United Arab Emirates

**Keywords:** Bronchiolitis, Respiratory syncytial virus, Hyponatremia, Infants, Hospitalization, Prematurity, Viral diseases

## Abstract

**Background:**

Bronchiolitis is the commonest lower respiratory tract infection, found worldwide in children < 2 years of age. Over sixty percent of cases are caused by Respiratory Syncytial Virus (RSV). The disease is known to have significant morbidity, mortality and health care costs. Its seasonal variability, manifestations and complications vary between countries. The aim of this study was to determine the epidemiological and clinical characteristics of infants hospitalized with bronchiolitis in Al Ain City, United Arab Emirates.

**Methods:**

Retrospective observational chart review was made of an unselected cohort of infants ≤ 2 years admitted to the pediatric department of Tawam hospital over a 3-year period and discharged with the diagnosis of bronchiolitis. Epidemiological data and risk factors were analyzed.

**Results:**

RSV was the commonest pathogen (51%). Hospitalizations occurred year-round but increased significantly in December and January. The patients’ median age was 5.8 months with a male predominance (male:female ratio of 1.5:1.0). The mean age at admission was 6.6 months and presentation occurred, on average, 2.9 days after the onset of the symptoms. The majority (94%) had respiratory distress on presentation. Chest x-ray was performed in 80% of the patients. Most children received bronchodilator therapy and oxygen therapy was administered to 42%. The mean duration of hospital stay was 3 days.

**Conclusion:**

Bronchiolitis remains a common reason for hospital admission and carries significant morbidity. RSV is the primarily responsible virus for hospital admissions and morbidity.

A better understanding of the burden of bronchiolitis in our setting would enable better planning and use of hospital resources to minimize its short and long-term sequelae.

## 1 INTRODUCTION

Bronchiolitis is the commonest lower respiratory tract infection in children < 2 years of age and accounts for the majority of their physician visits and hospitalization during the winter season [1, 2].

The infection is mostly caused by Respiratory Syncytial Virus (RSV) in approximately 80% of cases [3]. Other causative viruses include influenza, parainfluenza, adenovirus and metapneumovirus [4, 5]. The most severe cases occur mainly in previously healthy term infants. Severe illness is associated with risk factors such as young age, prematurity, chronic lung disease, congenital heart disease, asthma, immune deficiency, deprivation and low socioeconomic status [6].

Some studies have shown that the type of virus is related to the outcome. Assessment of bronchiolitis severity through a combination of clinical symptoms and physical signs remains a standard measure in daily practice [6, 7]. Climate and enviro- nment appear to influence both its season and severity [8].

Bronchiolitis is diagnosed clinically by integrating characteristic but variable signs and symptoms across a broad age range, though the majority of cases occur in children under 1 year of age [9].

Typical symptoms are rhinorrhea, proceeding over 2 to 4 days to a characteristic harsh moist cough with pyrexia that is typically below 39°C, although fever above 38.5°C is seen in 50% of infants [10]. The ability to achieve adequate oral feeding declines as nasal obstruction with secretions develops and work of breathing increases. The time to peak symptoms of 4 days is associated with the peak in viral load varying from infant to infant [11].

In younger children (particularly <6 weeks of age), apnea may be a presenting sign, sometimes in the absence of other features of bronchiolitis [12]. Physical findings include an increased respiratory rate, chest recession, use of accessory muscles, hyperinflation, wheezing, crackles, and reduced arterial oxygen saturations. Physical findings vary depending on sleep state (and associated changes in tidal volume) [12].

Testing of nasal secretions for the virus may help consolidate the clinical diagnosis of bronchiolitis and inform health care logistics. Most commonly used, and with the highest precision, are Polymerase Chain Reaction (PCR) diagnostics for a range of respiratory viruses, but Point of Care (PoC) testing for a more limited range of viruses (most often RSV) is increasingly precise and cost-effective [13]

In UAE, the epidemiological data, viral etiologies and clinical description of this infection are scanty.

## 2 AIM

The aim of this study was to describe the epidemiological data, isolated organism, seasonal trends, clinical characteristics, and outcomes of children admitted with bronchiolitis in Al Ain city, United Arab Emirates.

Also, the objective involved determining the associated risk factors for hospitalization, seasonality, oxygen requirement, need for ventilator support, occurrence of other complications, and length of hospital stay.

## 3 METHODS

### 3.1 Study Design

This was a retrospective observational medical chart review of an unselected cohort of infants ≤ 2 years admitted to the pediatric department of Tawam hospital and discharged with the diagnosis of bronchiolitis over a period of 3 years (November 2008 to December 2011).

### 3.2 Study Location

The study location was Tawam Hospital, a tertiary care hospital in Al Ain City, United Arab Emirates.

### 3.3 Data Collection

Data were extracted through review of the electronic medical records of patients with a diagnostic coding of bronchiolitis on discharge (ICD codes 39710, 39713, 391714, 39719, 37892, 51206958, 803843, 3557595, 25236, 2732, 800541, 30590, 732839, J21.9, J21, J21.1, 2729, 2730, 71943, 803843). It included demographics, duration of illness before admission, and clinical features on admission. Fever was defined by a rectal temperature >38^0^C in infants less than 28 days and by tympanic thermometry for older children.

### 3.4 Inclusion and Exclusion Criteria

Inclusion criteria involved infants ≤ 2 years admitted with the clinical diagnosis of bronchiolitis on the admission made by the physician based on the presence of an upper respiratory tract infection (either by history or by cough or rhinorrhea on physical examination), tachypnea, hypoxia, cough, subcostal or intercostal retractions, nasal flaring, grunting, with wheezing and/or crackles on auscultation. Indications for admission included worsening of the respiratory status, decreased oral intake, and the requirement for oxygen or parenteral therapy. Only those with a discharge diagnosis of bronchiolitis were analyzed.

We excluded children with bacterial co-infections (meningitis, bacteremia, pyelonephritis or pneumonia), a history of cardiac, respiratory, endocrine disease or cystic fibrosis, renal, neurologic problems or medications likely to affect water and electrolytes balance, such as diuretics.

### 3.5 Laboratory Testing

As per departmental guidelines, nasopharyngeal aspirates were obtained on admission, using the specimen trap with Extra Transport Cap, and analysed by enzyme-linked immunoassay (ELISA) rapid antigen detection method for the respiratory syncytial virus (RSV) using the kit Tru RSV®, Meridian Bioscience, INC. Other viral respiratory pathogens (adenovirus, influenza A and B, parainfluenza 1, 2 and 3 viruses) were detected with the LIGHT DIAGNOSTICS™ SimulFluor® Respiratory Screen, EMD Millipore. Although not routinely performed, a Chest X-Ray (CXR) was ordered at the discretion of the attending physician.

### 3.6 Statistical Analysis

Proportions were compared with the Chi-squared test or the Fisher exact test when appropriate. The Student t-test was used to compare the means of normally distributed variables between the two groups. All statistical analyses were performed with Stata 13 (StataCorp. College Station, TX). A 2-tailed p-value was considered statistically significant if <0.05.

### 3.7 Ethical Approval

Approval was granted by the institutional review board (IRB 296/13) and the requirement for consent was waived as it was a retrospective study, and patient anonymity was preserved. The study was conducted in accordance with the ethical standards laid down in the 1964 Declaration of Helsinki and its later amendments.

## 4 RESULTS

### 4.1 Clinical and Demographic Characteristics

A total of 362 infants were enrolled in the study (Table **1**). The majority were males (n=230, 63.5%). The median age on admission was 5.8 months (range 0.1 to 5.1). Exclusive breastfeeding was the mode of feeding of 198 infants (55%), and 7.3% were on solids.

Fifty infants (13.8%) were ex-preterm, of whom six were born before 30 weeks of gestation. Palivizumab prophylaxis had been administered earlier to 12 infants (3.3%), including four with chronic lung disease and two with congenital heart disease. Eleven children had a medical history suggestive of reactive airway disease (3%) and six had underlying congenital heart disease (1.6%).

### 4.2 *Viral Detection* (Table **2**)

RSV was identified in 273 infants (75%), adenovirus in 24 (6%), influenza in 13 (3%), and other viruses in less than 1%. No viruses were identified in 50 patients (12%). Co-infection with RSV and Adenovirus and Influenza occurred in 8 and 6 patients, respectively.

### 4.3 Seasonality

**Fig. (1):**
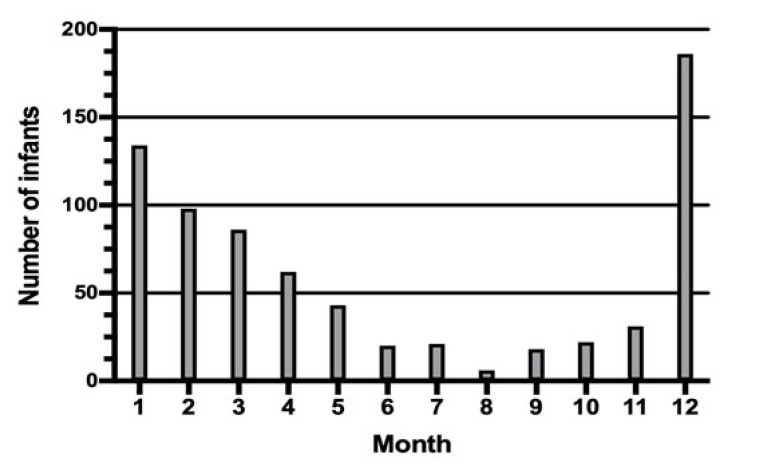
Number of infants admitted to hospital for bronchiolitis, by month, from November 2008 to December 2011.

Admissions with bronchiolitis occurred throughout the year (Fig. **1**), with a significant peak in December and January essentially related to RSV peaks (Table **2**). Adenovirus was identified mostly in May.

### 4.4 Clinical Characteristics

Most of the children presented on day 3 of the illness (range 2-5 days). On admission, 58% were having feeding difficulties, with associated vomiting in 38%. Fever was present in 34% of the infants, 44% had evidence of dehydration, 65% had tachypnea, 94% respiratory distress, 36% retractions, 2% grunting, and 4% cyanosis. Chest auscultation was normal in 25% of the children (Table **3**).

### 4.5 Hospital Admission

The mean time between the onset of symptoms and admission was 3.6 days (range 1 to 6). The mean ± SD duration of hospital stay for those who needed admission to the PICU was 4.2 ± 5.2 days (median 3, range 1 to 54 days).

### 4.6 Laboratory Investigations (Table **4**)

On admission, 17% of children had hyponatremia, with the lowest sodium concentration being 123 mmol/L. The median serum urea was 2.4 (range 0.1 to 23), 71% of children had a normal white cell count and 2 infants (0.5%) had thrombocytopenia.

### 4.7 Radiological Findings

Chest X-ray was performed in 80% of the patients. It was abnormal in 40% on presentation. A repeat CXR was required in 67 infants because of clinical worsening, at a median of 2 days after admission (range 0.5 to 9 days), and it was abnormal in 79% (Table **4**).

### 4.8 Management (Table **5**)

Oxygen supplementation was administered to 82% of the children, 80% received bronchodilators, 15.5% racemic epinephrine or hypertonic saline. Antibiotics were also administered to 17% and only 2% of admitted children received exclusively supportive treatment.

### 4.9 Severity of Illness and Complications

Complications during hospitalization (Table **6**) included apneas in 17 patients (4.5%), cardiac complications in 5 patients (1%) and encephalopathy in 1 infant. 14 infants had pneumonia (3.86%), 16 had acute otitis media (4.4%) and one developed supraventricular tachycardia (0.27%). Hyponatra- emia was found in 18 patients (5%).

Forty children (11%) required admission to PICU (Table **7**). In 25 patients (63%), CPAP was required. Additional oxygen administration by high flow nasal cannula was the only therapy administered to 6 children (15%). Mechanical venti- lation was required in 9 children (22%). No death occurred in infants while in the hospital.

## 5 DISCUSSION

Our study focused on healthy infants and all of those with other co-morbidities were excluded from the study.

In our study, RSV was the main responsible organism as 273 patients (75%) had RSV as a sole organism or part of co-infection with other viruses. Other causative viruses included influenza, parainfluenza, coronavirus, adenovirus, metapneu- movirus, and rhinovirus. This was the same finding that was described in several other studies from UAE [14] and the Middle East [15-19], Europe [20-25], North America [26-28], and elsewhere [29-32].

RSV positive disease is usually associated with a more severe course, which occurs at a lower age, requires frequent oxygen administration, and is associated with a longer hospital stay, a higher rate of complications and higher hospital charges. Other authors have found that there is a significant association between the RSV genomic load and a longer duration of hospitalization [33, 34].

Male predominance (63.5% of patients) that we found in our stuy was the same as in reports on different age groups and from different countries [35]. A possible reason for this might be the disproportionally narrower peripheral airways in the younger males [36]. Similarly, males develop more severe disease and a higher rate of complications [37].

The infection occurred throughout the year, with peaks during the winter months, and no signification difference was found with respect to the geographic areas. The temperature during these months in UAE ranges from 20–28^0^C during the day and 10–18^0^C during the night. It seems, therefore, that the virus may respond to a temperature change rather than to an absolute range of lower temperature [8, 38, 39]. Humidity is positively associated with RSV activity, with more RSV infection occurring when humidity increases. The average humidity in UAE is around 50% over the year; on average, December is the most humid month and May is the least humid month.

Many studies demonstrate the effects of relative humidity on pediatric respiratory tract infections. In tropical and subtropical areas (*e.g.*, Singapore, Malaysia), it has been suggested that high humidity and temperature prolong the activity and stability of microorganisms in large-particle aerosols, and permit year-round transmission of the microo- rganism [40]

Our study showed that most of the patients presented with respiratory distress as also described in previous literature [41, 42]; abnormal chest auscultation was found in 75% of patients in our study which may be due to admitting mild cases with scanty manifestations.

The sensitivity and specificity of immunofluorescence for the diagnosis of RSV infection have been reported to be in the range of 70–90% [43]. The lack of identified viruses in 50 of our patients could be explained by the late sample collection in the course of the illness when the viral load had already decreased.

There are several predictors of disease severity that have been described in the literature, such as male sex, young age, birth in the first half of the RSV season, daycare attendance, lack of breastfeeding, chronic medical conditions, smoke exposure, and house-hold crowding or siblings [44].

The mean hospital duration of stay in our patients was 3.6 days for patients admitted to the regular ward and 4.2 days for those admitted to PICU. Disease severity and LOS usually depends on the clinical characteristic of the illness [45]. The higher the viral load is, as quantified by RT-PCR, the more severe the infection is [46].

Management of bronchiolitis is usually symptomatic as recommended by most of the guidelines; however, most of our patients received bronchodilators and different kinds of nebulizers. For this, new guidelines and audits need to be performed on the departmental level [47-49].

No therapies have received support across all guidelines except for the use of supplemental oxygen. Chest physiotherapy does not speed up recovery. Antibiotics, though still widely used, are of no benefit in bronchiolitis [50].

Some of our patients received different treatment modalities like bronchodilators. Bronchodilators are less likely to be recommended in more recent guidelines, and the theory that they may be of greater benefit in infants more likely to develop asthma has been refuted [51, 52]

The limitation of our study is its retrospective nature. In addition, concomitant bacterial infection and viral genomic load have not been measured.

We recommend the design of a prospective, multi-site surveillance program of viral bronchiolitis in such a warm, desert climate. Guidelines based on evidence and local data are crucial to be established and followed to improve the outcomes of this disease.

## CONCLUSION

Bronchiolitis remains a common reason for admission to hospitals with significant morbidity and mortality. RSV has been found to be the main responsible virus.

A better understanding of the burden of bronchiolitis in our setting would enable better planning and use of hospital resources to minimize the short and long-term sequel of the disease.

## Figures and Tables

**Table 1:** Demographics data of 362 infants admitted to hospital for bronchiolitis.

-	Total	RSV Positive	RSV Negative	P Value
Infants	362	190 (52)	176(48)	NA
Mean (SD) number of days between the onset of symptoms and admission	2.9 (1.2)	2.8 (1.2)	2.7 (1.6)	0.6
Males	230 (63.5)	158 (83)	25 (14.2)	0.3
Mean age (SD) in months	6.6 (5.6)	6.8 (5.8)	5.1 (4.2)	0.08
Exclusively breastfed	198 (54.6)	64	134	0.6
Fed solids	23 (7.3)	20	3	0.6
Preterm < 38 weeks	50 (13.8)	45	5	0.5
Palivizumab prophylaxis	12 (3.3)	11	1	0.4

Number (%) unless stated otherwise. NA - not available.

**Table 2:** Number of viruses isolated in 362 infants admitted to hospital for bronchiolitis.

**Month**	**Respiratory Syncytial Virus**	**Adenovirus**	**Influenza A or B**	**Total Viruses Isolated**
January	62	0	1	63
February	37	3	2	42
March	17	3	3	23
April	8	3	1	12
May	4	6	0	10
June	4	3	0	7
July	6	1	0	7
August	2	0	0	2
September	7	0	0	7
October	10	0	1	11
November	17	1	0	18
December	99	4	5	108
**Total**	273	24	13	310

**Table 3:** Frequency (%) of the clinical features at presentation (by descending order of frequency) of 362 infants admitted to hospital for bronchiolitis.

Respiratory Distress	94
Abnormal chest auscultation	75
Tachypnea	64
Feeding difficulties	58
Dehydration	44
Hypoxemia (O2 saturation < 92% in air)	41
Vomiting	38
Chest Retractions	36
Fever (temperature more than 38^0^C	34
Cyanosis	4
Grunting	2

**Table 4:** Percentage of abnormal investigation results in 362 infants admitted to hospital for bronchiolitis.

Hyponatremia (serum Na <135 mEq/L)	17%	
Mean (range) serum urea mmol/L	2.4 (0.1-23)	
Elevated WBC count 10,000 cells per microliter (cells/mcL)	79%	
Thrombocytopenia (Platelet counts of 500 x 10^9^/L)	0.5% (2 infants)	
Abnormal CXR	40%	
Repeat CXR	67 infants Median 2 days (range 0.5-9) after admission Abnormal in 79%	

**Table 5:** Management and outcome by descending order of frequency (expressed as a percentage) of 362 infants admitted to hospital for bronchiolitis.

Oxygen Administration		82
Beta-2 agonists inhalation		80
Racemic Epinephrine		15.5
Hypertonic saline nebulizer		27.6
0.25% NaCl intravenous maintenance fluids		80
0.45% NaCl intravenous maintenance fluids		19.6
Antibiotics		17

**Table 6:** Complications observed in 362 infants admitted to hospital for bronchiolitis.

Apneas	17 (4.5%)
Cardiac complications	5 (1%)
Encephalopathy	1 (0.2%)
SVT	1 (0.27%)
Hyponatremia during hospitalization	18 (5%)

Number (%) unless stated otherwise

**Table 7:** Intensive care admissions in 362 infants admitted to hospital for bronchiolitis.

Admission to PICU	40 (11)
CPAP	25 (63)
Mechanical ventilation	9 (22)

## LIST OF ABBREVIATIONS

CPAP: = Continuous Positive Airway Pressure
CHD: = Congenital Heart Disease
LOS: = Length of Stay
PICU: = Pediatric Intensive Care Unit
RSV: = Respiratory Syncytial Virus
SD: = Standard Deviation
